# Examining the Role of Telemedicine in Diabetic Retinopathy

**DOI:** 10.3390/jcm12103537

**Published:** 2023-05-18

**Authors:** Matthew R. Land, Parth A. Patel, Tommy Bui, Cheng Jiao, Arsalan Ali, Shadman Ibnamasud, Prem N. Patel, Veeral Sheth

**Affiliations:** 1 Department of Ophthalmology, Medical College of Georgia, Augusta University, Augusta, GA 30912, USApapatel1@augusta.edu (P.A.P.);; 2 Burnett School of Medicine, Texas Christian University, Fort Worth, TX 76129, USA; 3 Department of Ophthalmology, University of Texas Southwestern Medical Center, Dallas, TX 75390, USA; 4 Department of Ophthalmology, University Retina and Macula Associates, Oak Forest, IL 60452, USA

**Keywords:** diabetic retinopathy, telemedicine, artificial intelligence, teleophthalmology, multidisciplinary cooperation

## Abstract

With the increasing prevalence of diabetic retinopathy (DR), screening is of the utmost importance to prevent vision loss for patients and reduce financial costs for the healthcare system. Unfortunately, it appears that the capacity of optometrists and ophthalmologists to adequately perform in-person screenings of DR will be insufficient within the coming years. Telemedicine offers the opportunity to expand access to screening while reducing the economic and temporal burden associated with current in-person protocols. The present literature review summarizes the latest developments in telemedicine for DR screening, considerations for stakeholders, barriers to implementation, and future directions in this area. As the role of telemedicine in DR screening continues to expand, further work will be necessary to continually optimize practices and improve long-term patient outcomes.

## 1. Introduction

Diabetic retinopathy (DR) is a microvascular, ocular complication of diabetes mellitus that is prevalent across the globe. Approximately 463 million people were estimated to be affected by the condition in 2019, a figure projected to expand to 700 million by 2045. In particular, DR is expected to disproportionately affect populations in the Middle East, North Africa, and the Western Pacific [1]. Contending with the long-term effects of this disease constitutes a substantial monetary investment. A relatively recent economic model of patients with DR in Indonesia, a nation with a considerable burden of disease, identified that the summative healthcare costs to the government for DR comprised approximately 2% of the state budget in 2017. Much of this expense was attributable to treatments for patients with more advanced DR such as vitrectomy and intravitreal injection [2].

Preempting these costs requires the implementation of strategies to screen patients with diabetes mellitus in order to detect disease early. Traditional detection and management of DR depend upon in-person retinal examination, with professional ophthalmic care providers performing ophthalmoscopy or imaging the retina to capture damage to underlying vasculature. The sustainability of in-person universal screening, however, is questionable [3]. As the number of individuals with diabetes increases, an exponentially rising number of hours will be required from ophthalmologists to examine each patient once annually [4,5]. Financially, expenditures may surmount hundreds of millions of dollars, particularly when accounting for the hidden costs of healthcare including absences from work and transportation [6,7]. Other factors compounding these concerns include psychological and social considerations e.g., age, poor health literacy, and limited health insurance [8,9,10]. As such, despite concerted efforts to increase access to screening from various agencies and organizations, a substantial proportion of patients with diabetes mellitus do not receive annual eye examinations [11,12]. Accordingly, novel protocols integrating teleophthalmology are necessary to effectively address the unmet need of evaluating patients with DR.

Telemedicine is the distribution of remote healthcare services to physically separate clinical providers and patients. One of the first documented uses of telemedicine was in the 1950s, when psychiatric consultations were delivered via closed-circuit television at the Nebraska Psychiatric Institute and Norfolk State Hospital [13]. Since then, technical advancements have facilitated rapid changes in this space, thereby improving the efficiency of this healthcare delivery modality [14,15]. Internationally, healthcare systems have increasingly adopted telemedicine to deliver more accessible, cost-effective care, especially among remote or underserved communities [16]. These considerations are particularly pronounced in the context of the recent COVID-19 pandemic that mandated restriction of person-to-person contact between healthcare providers and patients. The resulting decreased access to medical services across all societal strata necessitated rapid innovation in order to enable clinicians to provide continuing effective healthcare for their patients. In this setting, for a smaller specialty such as ophthalmology, where a relatively limited number of practitioners are delivering ocular healthcare to a remarkably larger number of patients [17], telemedicine became progressively more essential for the successful provision of medical coverage [18,19,20]. Evidence of this phenomenon is found in an investigation by Portney and colleagues, who reported that a peak of 17% of all ophthalmic encounters in the U.S. state of Michigan were comprised of telehealth visits in early April 2020 [21].

Nonetheless, there remains substantial potential for the expansion of teleophthalmology services. DR screening, specifically, is an area where teleophthalmology can be effectively leveraged to reduce the economic, temporal, and societal burden associated with the management of the condition. As discussed previously, the current literature indicates that the increasing prevalence of DR will soon result in demand for screening vastly exceeding the capacity of eye care specialists to provide timely in-person evaluation for patients [4,5]. Teleophthalmology represents one potential solution to the aforementioned concerns as it is accessible and cost-effective for patients while possessing a sensitivity and specificity largely comparable to those of traditional clinical exams [22]. Given this utility, we review the integration of teleophthalmology into DR management and the associated advantages, considerations, and trends.

## 2. Accuracy and Reliability of Teleophthalmology

To decrease rates of preventable vision loss, ophthalmologic telemedicine should both reliably diagnose early disease and appropriately refer high-risk patients for more-intensive follow-up. For tele-retinal imaging (TRI) to be considered a viable screening tool, it must accurately determine the likelihood for a patient to have DR [23]. A recent meta-analysis of multiple large-scale studies reported that TRI screening programs detect threshold-level DR with high sensitivity (91% (95% confidence interval, CI 0.82–0.96)) and specificity (88% (95% CI 0.74–0.95)), figures comparable to the traditional clinical examination [24]. A more granular meta-analysis of 33 articles involving more than 10,000 patients identified that teleophthalmology had a cumulative sensitivity for high-risk PDR of 76% (standard deviation (SD) ± 31%) and specificity of 94% (SD ± 9%) relative to gold-standard ophthalmoscopy [25]. For low-risk PDR, the mean sensitivity was 75% and the mean specificity was 98% (SD ± 2%). For severe NPDR, the mean sensitivity was 42% (SD ± 27%) and the mean specificity was 94% (SD ± 1%). Sensitivity and specificity were 87% and 91%, respectively, for detecting the absence of retinopathy.

These findings have been replicated across various populations and settings using a myriad of protocols and screening devices. A focused investigation employing mobile screening devices noted that telemedicine with handheld nonmydriatic images could adequately examine and accurately diagnose fundus pathology [26]. A total of 200 patients were evaluated on-site by an ophthalmologist. Additionally, fundoscopic images were captured using both handheld nonmydriatic and conventional fundus cameras. All photographs were randomized and presented digitally to two remote ophthalmologists. Although the nonmydriatic camera provided lower image quality and a smaller field of view (FOV), nearly 80% of the images were classified as excellent (22.7%) or good (55.7%), indicating that less than 50% of the fundus field was blurred and an appropriate diagnosis could be made. The diagnosis agreement rate with the on-site exam was comparable between the handheld nonmydriatic and conventional fundus images at 90.7% and 95.2%, respectively.

Yeh and colleagues reported similar findings in their investigation of the viability of handheld devices for teleophthalmology screening [27]. Eighty-eight patients underwent comprehensive eye examinations, including an indirect ophthalmoscope exam with a retinal specialist. A total of 176 fundus images were captured. Deidentified photos were remotely reviewed by two retinal specialists and independently graded according to the same criteria as the on-site indirect exam. Referral recommendations for each participant were based on the American Academy of Ophthalmology (AAO) Practice Pattern Guideline [28]. Over 95% of the images were of acceptable or ideal quality for assessing details and emergent findings of the fundus based on the modified FOTO-ED scale [29]. Relative to the gold-standard indirect fundus exam, there was considerable agreement between the two reviewers’ assessments and the on-site diagnosis (88.6–89.8% with a Cohen’s k coefficient of 0.78–0.80). Sensitivity and specificity were 78% and 99%, respectively. Likewise, the two reviewers averaged 94.8% (k = 0.81) agreement with the on-site referrals. Overall, telemedical screening using handheld retinal imaging created sufficient quality fundus photographs that can be utilized for referral-driven diagnosis comparable to standard slit lamp ophthalmoscopy.

Date et al. conducted a broader population-level assessment of the accuracy of teleophthalmology screening for referable DR grading with 24,138 Harris Health System (HHS) patients [30]. Where appropriate, low-income diabetic patients were referred by their primary care provider (PCP) to 1 of 12 outpatient sites to obtain single-field 45-degree nonmydriatic color fundus imaging. Images were uploaded to a HIPAA-compliant server and assessed by a trained ophthalmologist following the International Clinical Diabetic Retinopathy Disease Severity Scale with follow-up recommendations for patients with severe non-proliferative diabetic retinopathy (NPDR), proliferative diabetic retinopathy (PDR), or significant diabetic macular edema (DME). On the basis of these TRI readings, 1767 patients were included for dilated fundus examination (DFE) in the clinic. Those with lower levels of DR were asked to return in six months to one year. The positive predictive value for detecting referable-level DR (severe NPDR or PDR) was 71.3%. When comparing the DR severity grading on TRI with that of in-clinic DFE, there was moderate agreement, with a weighted k coefficient of 0.45. Nonetheless, a significant portion of TRI grades for severe NPDR (90%) were within one DR severity level of the clinical examination grade. As for PDR, 78% of TRI grading was an exact match, and 7% was within 1 severity level. Among those with >2-step discrepancy in DR grading, 69.3% were over-estimated by TRI. Overall, these accuracy levels reassuringly highlighted that TRI could assign correct DR severity and ensure appropriate follow-up recommendations, even when screening at a population level.

It should be noted, however, that the results of teleophthalmology programs are dependent on the eye care providers serving as the readers, with individual experience levels varying substantially. Using a set of masked images, Liu et al. assessed the variability between an optometrist, a general ophthalmologist, and a fellowship-trained retina specialist for tele-retinal evaluation of DR [31]. Among those cases deemed gradable (*n* = 65), there was substantial agreement on the absence of any retinopathy (88% (SD ± 4.6%)), presence of moderate non-proliferative or worse retinopathy (87% (SD ± 3.9%)), and macular edema (99% (SD ± 0.9%)). All cases (*n* = 7) of clinically significant DR identified by the retinal specialist were similarly identified by the other two readers. There was comparable agreement with referral recommendations for urgent (one month), nonurgent (two to six months), and routine (one year) follow-ups. Conversely, agreement was limited regarding the presence of referable nondiabetic eye pathology (61% (SD ± 11%)). Furthermore, the intrareader agreements among providers were variable for diagnoses and dispositions, with the general ophthalmologist and optometrist having a lower degree of intrareader agreement (60%) than the retina specialist (90%) [31].

## 3. Access to and Cost-Effectiveness of Teleophthalmology

Teleophthalmology is becoming more widespread due to its ability to provide timely and appropriate treatment to patients who may not be able to attend appointments as frequently, particularly those residing in rural areas. Because DR is asymptomatic in its early stages, patients with diabetes mellitus must be screened regularly if they are found to have retinopathy [32]. Utilization of teleophthalmology may improve multiple aspects of DR management, including access to treatment, rates of follow-up, early treatment, and cost of care.

Using a pretest–posttest model, Daskivich and colleagues examined the implementation of teleophthalmology to improve services rendered to patients of the Los Angeles County Department of Health Services. At baseline, the average wait time for ophthalmology screening for DR in the Los Angeles County safety net was more than 8 months for underinsured patients [33]. Following the institution of a primary-care-based teleretinal DR screening program, during which 21,222 patients were evaluated over 2 years, screening rates improved by 16%, and median wait times were reduced from 158 days to 17 days. Their findings suggested that the U.S. safety net for underinsured and uninsured populations would reap substantial benefits by investing in telehealth programs that could broaden access to care.

Teleophthalmology is similarly more cost-effective in a myriad of circumstances. A DR screening initiative in the Bronx, the most diverse and lowest-income borough of New York City, illustrated both the cost-effectiveness and potential profitability of such programs [34]. Treatment of DR-associated pathology identified on screening resulted in a gain of 9 quality-associated life years (QALY) in addition to USD 49,052 of savings. Furthermore, on the basis of the reimbursement rates from the Centers for Medicare and Medicaid Service (CMS), treatment of DR-related pathology generated USD 208,535 in revenue [34]. In Southeastern Brazil, a similarly diverse setting, an assessment of the economic viability of DR teleophthalmology screening revealed an average cost reduction of USD 28.76 per patient. The authors calculated that achievement of the break-even point (where revenue and cost are identical) would require a mere 112 exams per month or 1344 exams per year [35]. Rachapelle et al. evaluated the cost-effectiveness of implementing various DR teleophthalmology screening intervals in India, which possesses a relatively large rural population. Utilizing the World Health Organization definition of cost-effectiveness, they determined that teleophthalmology for DR screening was more cost-effective relative to no screening if the frequency interval of examination was greater than one year [16].

Greater multidisciplinary cooperation may further expand upon the cost-effectiveness of teleophthalmology screening for DR. Although some PCPs and optometrists may recognize evidence of the disease, there are circumstances where it is misdiagnosed or underdiagnosed, thereby conferring a greater lifetime cost to the healthcare system and the patient. A relatively recent economic analysis evaluated the cost-effectiveness of a pilot DR teleophthalmology screening program by employing a decision tree model [36]. Images were captured by PCP, uploaded to a standardized system, and then graded by a retina specialist. The costs of screening 566 patients via the teleophthalmology pilot program were compared against in-person screening. Findings indicated that the screening program accurately diagnosed more patients (496 vs. 247) and was cost-saving (USD 82.4 vs. USD 237.8). In effect, teleophthalmology can differentiate patients with DR who can be screened remotely (minimal to mild DR) versus those who need comprehensive evaluations (moderate to severe DR), enabling more efficient use of specialists’ time and increased access to those requiring specialty visits [37,38].

## 4. Patient Perceptions of Teleophthalmology

Patient recognition and acceptance of teleophthalmology as an effective tool for early screening of DR is a key factor in its success. Overall, patients generally possess a favorable perception of telemedicine, in part due to its convenience as a method for obtaining DR screening. An investigation evaluating the factors that may impact patients’ attitudes toward telemedicine found that most patients viewed the service as convenient [39]. Other factors likewise influenced patient perceptions of teleophthalmology. Patients with a larger number of ocular comorbidities and those with limited access to in-person care were more likely to view teleophthalmology as a convenient alternative to traditional screening methods. Alternatively, patients who valued their patient–physician relationships or had long-standing diabetes mellitus were less receptive to the technology.

The appeal of telemedicine screening is additionally influenced by patient satisfaction with the experience. A study conducted on a remote island with indigenous populations noted that approximately 97.5% of patients (*n* = 90) were satisfied with the utilization of the telemedicine screening program and were receptive to future participation in telemedicine encounters [27]. While this high degree of satisfaction among these specific participants may be surprising considering their background, the findings corroborate the concept that patients lacking eye care resources and residing in a locale with a high prevalence of ocular diseases have better perceptions of the value of telemedicine screening for DR (and correspondingly, higher satisfaction).

Lastly, DR telemedicine screening has been demonstrated to be a valuable and effective tool for patient education and early detection of ocular diseases. According to a survey of patients who participated in the Toronto Teleretinal Screening Program, 71.7% of individuals who had never been screened previously revealed a lack of awareness of DR. Nonetheless, 91.6% attested to the usefulness of the screening services, further demonstrating the efficacy of telemedicine screening for detecting DR [40]. A study by Ramchandran et al. showed that 35% of participants cited the ability to detect diseases early and stay informed about their eye health as reasons for undergoing teleophthalmology exams during primary care visits [41]. The addition of these screening methods provides a sense of reassurance to patients that there are no potentially vision-threatening conditions present, as well as the opportunity for early detection of DR so that treatment can be pursued to prevent vision loss. Telemedicine screening further offers the opportunity for patients to receive personalized education from their healthcare providers about their eye health through accessible retinal images. Conversely, concerns remain, particularly among older patients, regarding the level of expertise and thoroughness of teleophthalmology exams provided by primary care providers.

## 5. Barriers to the Implementation of Teleophthalmology

### 5.1. Cost and Reimbursement

Importantly, implementing telemedicine for DR screenings requires a substantial initial investment to acquire the necessary hardware and software [42], although handheld and phone-based fundus cameras may serve as cheaper alternatives to tabletop cameras [43]. Additional costs are associated with training new staff and requiring auxiliary tasks to support screening protocols, although retraining current staff, leveraging artificial intelligence (AI), and optimizing workflow can mitigate financial burdens related to personnel [44]. Furthermore, while teleophthalmology may traditionally be considered a cost-effective long-term alternative to in-person DR screenings [45], many studies evaluating cost-effectiveness may not be generally representative of U.S. healthcare systems [44]. Indeed, studies by groups such as Kirklizar et al., who evaluated the telemedicine screening program used by the Veterans Health Administration department, failed to account for billing and reimbursement, issues that remain major barriers to the widespread implementation of teleophthalmology screenings for DR [46].

Historically, telemedicine reimbursement has been a barrier to implementation within the U.S., as providers may be hesitant to offer these services due to concerns about adequate compensation [42]. However, the COVID-19 pandemic has resulted in a dramatic improvement in telemedicine reimbursement as a consequence of the Coronavirus Preparedness and Response Supplement Appropriations Act of 2020 [47]. Despite this progress, work by Lee et al. reinforced that reimbursement for teleophthalmology screening for DR will remain an obstacle for the foreseeable future [48]. Their investigation examined trends in reimbursement for the Current Procedural Terminology (CPT) codes used for remote retinal imaging: 92227, 92228, and 92250, from 2011 to 2021. The analysis showed a decrease in the proportion of claims paid for remote imaging over the study period, especially 92227, which is designated for screening diabetic patients without eye diseases. Of note, the percentage of claims paid for patients with Medicare Advantage was significantly lower than Commercial Insurance for the entire time period studied. Furthermore, the reimbursement for the least specific code, 92250, had risen in comparison to 92227 and 92228, incentivizing inappropriate coding for screening patients without known diabetic eye disease. The decline of and inconsistencies within medical coverage were cited as barriers to the widespread adoption of remote retinal imaging. The impact of these obstacles is magnified by the finding that patients most frequently report out-of-pocket costs as a barrier to teleophthalmology [49]. The adoption of cost-effective screening technologies and payment reform offer potential solutions to the economic barrier of implementing telemedicine screenings [42,50].

### 5.2. Image Quality of Non-Mydriatic Eyes

Currently, ocular dilation is not universally plausible, particularly in settings without ophthalmic specialists, thereby restricting the inherent quality of images captured for examination. Although handheld non-mydriatic fundus cameras offer a potential solution to circumvent this concern by reducing cost and increasing portability and ease of use [43], notable limitations are present. Despite technical advancements, non-mydriatic fundus cameras continue to capture a notably greater proportion of ungradable images versus the gold standard of mydriatic table-top cameras [44,51]. In an analysis of 700 patients in Sri Lanka with diabetes mellitus, Piyasena and colleagues compared the quality of images acquired through a handheld non-mydriatic digital retinal camera (Visuscout 100^®^-Jena, Germany) before and after pupillary dilation. They identified that, relative to 12.8% of images acquired after pupillary dilation, 43.4% of images acquired through non-mydriatic imaging were ungradable (<50% gradability) [52]. A similar comparative assessment of 155 subjects with and without diabetes mellitus found 17–18%, 8–12%, and 5–7% of images obtained by non-mydriatic Smartscope, mydriatic Smartscope, and mydriatic table-top camera (TRC—50DX [Topcon Corporation, Tokyo, Japan]), respectively, to be ungradable [53]. To date, no large clinical trials have illustrated significant advancements in non-mydriatic cameras to eliminate the disparity in image quality.

Ungradable images necessitate further in-person examination by ophthalmic specialists, leading to a reduction in the cost-effectiveness and efficiency of teleophthalmology. While the implementation of protocols preemptively integrating selective mydriasis may increase overall image gradability rates [54], such algorithms themselves present concerns, particularly with logistics and patient perceptions of DR screening [49].

Ultrawide-field (UWF) retina cameras can resolve some of the image quality issues associated with standard non-mydriatic fundus cameras. Relative to the 30–50° field of view of non-mydriatic fundus cameras, UWF retina cameras capture a 200° field of view, representing approximately 82% of the retinal surface. As such, UWF retina cameras are able to achieve lower ungradable rates and quicker acquisition times without dilating drops [55]. Nevertheless, their expense is significantly greater than non-mydriatic fundus cameras [44]. Additional limitations include image artifacts (e.g., opacities, eyelashes), peripheral distortion, and requisite technician experience. Further improvements in technology are required to address the above concerns. In the interim, it may be prudent for lower-resource countries to adopt non-mydriatic cameras and for higher-resource countries to utilize UWF lens [56].

### 5.3. Implementation into the Clinical Workflow

Widespread integration of telemedicine into the typical clinical routine can be challenging, as it requires providers and staff to adapt to new technology and alter already complex workflows, as evidenced by the experiences of clinicians during the COVID-19 pandemic [57]. Indeed, in the realm of teleophthalmology, Wandy and colleagues investigated the implementation of DR screening in three primary care clinics. Though all clinics had increased DR screening via the program, the largest increase in DR screening (24%) was observed in the practice with an optimized workflow for telemedicine. The aforementioned clinic used medical assistants to direct patients with diabetes to receive an eye exam via a standing order, thereby illustrating the importance of optimized workflows for teleophthalmology programs [58].

Towards that end, the American Telemedicine Association (ATA) and the AAO have provided guidelines for teleophthalmology programs; however, each health system must adapt these recommendations to cohere with their unique needs and resources [44]. Long term, developing effective and efficient workflows is imperative to decrease the burden and prevent the abandonment of screening programs. A qualitative study by Bouskill et al. illustrated the workflow interruptions that may stem from the additional strain on staff introduced by DR teleophthalmology screenings [59]. The predominant issues centered around scheduling patients, coordinating follow-up care and treatment, and improving adherence to diabetes treatment. While the implementation of teleophthalmology increased the number of workarounds or deviations from outlined workflows by medical staff, the authors noted these workarounds may prove foundational for the design of solutions to prevent additional workflow disruptions [59]. Further interrogation of existing workflows and systematic attempts to bolster resources will be essential for the continued improvement of teleophthalmology infrastructure across clinical practices.

### 5.4. Healthcare Personnel Training

Because of the relatively limited number and maldistribution of ophthalmologists globally [60], non-ophthalmologist healthcare personnel will need to be recruited to effectively screen patients. Unfortunately, widespread training of these providers remains an obstacle. Among PCPs, some of the identified primary barriers to the implementation of teleophthalmology include difficulty recognizing appropriate timing for diabetic eye screening, unfamiliarity with teleophthalmology, and lack of access to patient ocular health records [49]. Similar findings were reported in a qualitative assessment of nurse and nurse practitioner attitudes toward the implementation of teleophthalmology in their primary care clinics [61]. Integration of alerts in the electronic health record (EHR) system regarding the scheduling of follow-up appointments, further training on the use and purpose of teleophthalmology, and enhanced PCP access to patient ocular health records could reduce these consternations [49].

Shortages of ophthalmic care providers to grade pictures additionally limit the widespread implementation of telemedicine screening for DR [44]. Overarchingly, the literature suggests that 17% of the global population has access to less than 5% of ophthalmologists [60]. Indeed, Egunsola et al. noted the need for additional healthcare personnel training as a recurring theme in their systematic review of factors influencing uptake of DR teleophthalmology screening [62]. In particular, lack of experience among screeners and graders is cited as a common concern in regions such as sub-Saharan Africa [63]. Possible interventions include partnering with other healthcare systems (domestic and international) and commercial reading centers to increase the reading capacity of a screening program. AI remains another option to decrease or entirely eliminate the burden on readers [64]; however, this solution presents itself with an entirely new host of barriers related to implementation and acceptance [65]. Multiple studies have also evaluated the potential for non-specialists to grade images, revealing acceptable levels of agreement between them and eye care providers, with exceptions [31,66,67]. Unfortunately, these studies are limited by small numbers of trainers. Larger clinical trials are necessary to evaluate the plausibility of introducing non-specialists to the reading pool.

### 5.5. Privacy and Security of Healthcare Data

Privacy and data security were identified as major barriers to the implementation of telemedicine overall in more than half of the included studies in a systematic review by Ftouni et al. [68]. Although a necessary adjustment during the COVID-19 pandemic, the relaxation of the enforcement of the Health Insurance Portability and Accountability Act (HIPAA) reduced the protections afforded to patient data. Specifically, these modifications enabled platforms such as Zoom and Skype to be utilized in telemedicine [69]. Even with the strict regulations in place prior to the pandemic, 90% of healthcare providers reported encountering a data breach in the past [70]. Therefore, it is important to recognize that platforms and websites affiliated with the provision of telemedicine may collect or leak information, thereby compromising patient–physician confidentiality and posing an ethical challenge [71].

Because compromises to patient privacy and data pose serious threats in the implementation of telemedicine, prioritizing the security of healthcare data by implementing precautions is crucial to ensuring the long-term success of teleophthalmology screenings for DR. To preserve confidentiality, both informed consent and education of patients regarding cybersecurity risks are needed. Ophthalmology is no exception to this practice, as a statement issued by the AAO in 2018 highlighted compliance with HIPAA and secure transmission of data as necessary considerations for teleophthalmology [72]. Improving security can involve a multitude of precautions, as outlined in the Ophthalmic Digital Health Workshop in 2019 [73]. Robust measures such as utilizing cloud-based storage, mandating strong passwords, and providing basic security training to personnel represent just a few of the steps to minimize unauthorized access or disclosure and safeguard patient privacy. More broadly, implementing uniform guidelines (e.g., General Data Protection Regulation in the European Union) will likely be necessary to ensure maximum data protection across healthcare systems providing DR teleophthalmology screening [74].

### 5.6. Broad Ethico-Legal Challenges

Apart from data protection and security, telemedicine faces numerous ethical dilemmas. One of the hurdles of telemedicine is to ensure that patients have equal access to healthcare services, irrespective of their geographic location, financial circumstances, or socioeconomic status. Preservation of equity becomes more complex in locales with inadequate technology and internet infrastructures, resulting in an unequal distribution of telemedicine services and widening the existing disparity in healthcare access. For example, one report broadly examining telemedicine usage for chronic disease management during the COVID-19 pandemic observed that Blacks and Latinx had a decrease in the proportion of patient visits following the introduction of telemedicine [75]. Comparably, an analysis of ophthalmic encounters at one academic medical center found Blacks (odds ratio (OR), 0.45 (95% CI 0.32–0.62)) and Latinx (OR, 0.56 (95% CI 0.37–0.83)) had a substantially reduced likelihood of completing a telemedicine appointment [76]. This issue can partially be linked to differences in digital literacy and access to relevant resources, otherwise denoted the digital divide [77]. Thus, offering telehealth services is alone insufficient to ensure equitable access to ocular healthcare. Resolving this digital divide will necessitate the implementation of community-driven outreach programs, increased technical investment, and establishment of codes of conduct and comprehensive regulations that will be appropriately followed by involved parties.

Unfortunately, given the recent emergence and rapid advancement of teleophthalmology services, no global ethico-legal standards have been established specifically for teleophthalmology. Rather, current regulations are guided by local or national laws for telemedicine, which may be minimal or nonexistent [78]. Indeed, there remains a dearth of literature regarding laws and regulations for telemedicine due to the diverse approaches (or lack thereof) adopted around the world [74,78]. This variation in legal standards could impair the handling and security of protected health information, particularly in competitive markets. Of the few studies examining legal standards, the majority recommended more unified legislation in telemedicine [74]. Collaboration between stakeholders (administrators, patients, ocular healthcare providers, researchers, etc.) is necessary to create guidelines specific to teleophthalmology [79].

These regulations are particularly necessary, provided that the technical barriers to implementing teleophthalmology for DR screening (as discussed in Section 5.2) can further create ethical challenges. Poorer image quality of images captured outside of the clinic relative to in-person examination presents the risk of obscuring the correct diagnosis from health professionals involved with teleophthalmology screenings [79]. The resulting misdiagnosis can lead to readers being held medically liable for situations without defined legal statutory clauses. Consistent with this concern is the finding of one analysis that 68% of telehealth-related malpractice lawsuits were the product of a failed diagnosis [80]. A more contemporary investigation in ophthalmology reported similar results, with 61.1% of cases alleging misdiagnosis [81]. This uncertainty can affect the willingness of ocular healthcare professionals to adopt teleophthalmology practices, an attitude widely mirrored among other medical practitioners [82]. Indeed, a relatively recent survey of 242 physicians in Pakistan revealed that approximately 69% agreed or strongly agreed that regulations were insufficient to guard against malpractice [83]. Therefore, creating more well-defined standards is warranted to accelerate the uptake of DR teleophthalmology screening.

## 6. Future Directions of Teleophthalmology

### 6.1. Artificial Intelligence

AI involves the use of machines and technology to automate intellectual tasks, such as learning, normally performed by humans [84,85]. Machine learning (ML) and deep learning (DL), both subsets of AI, are suited for higher-order processing, such as medical image interpretation [85]. In fact, both ML and DL algorithms have been utilized to evaluate glaucoma, age-related macular degeneration (AMD), and DR [84].

Recent research has illustrated the feasibility of AI platforms for the screening of DR. The AI algorithm DART analyzed 45-degree field fundus images (*n* = 1123 eyes) acquired via a non-mydriatic desktop camera. Images were independently classified as DR-positive or DR-negative by DART and an ophthalmologist. DART demonstrated a sensitivity of 94.6% (95% CI 90.9–96.9%) relative to human graders [86]. In another investigation, the FDA-approved IDx-DR AI system was compared with human grading (two retinal specialists) of seven-field UWF and full-field UWF images from 107 eyes of asymptomatic diabetic patients. IDx-DR AI showed great sensitivity, but poor specificity. Compared with seven-field UWF imaging, sensitivity was 100% (95% CI 83–100%), while specificity was 47% (95% CI 30–65%). Similarly, compared with full-field UWF imaging, sensitivity was 95% (95% CI 77–100%), while specificity was 47% (95% CI 29–65%) [87]. The Retinal Artificial Intelligence Diagnosis System (RAIDS) is a DL algorithm used in conjunction with evaluation by an ophthalmologist. Images graded as normal by RAIDS were left as such. However, abnormal images were then graded by an ophthalmologist. In combination with junior and senior retinal specialists, this approach provided a sensitivity of 90.6% (95% CI, 87.5–93.1%). Specificity for all groups was near 100%. Moreover, the combination approach required 75% less time compared to evaluation by an ophthalmologist alone [88].

### 6.2. Smartphone/Handheld Screening

AI has also been used to evaluate imaging captured with smartphone-based platforms. Photos obtained by the Remidio Non-Mydriatic Fundus smartphone application were analyzed through a smartphone AI algorithm for the presence of referable DR (RDR). Results were compared against grading by two retinal specialists. For RDR, the smartphone app had a sensitivity of 100% (95% CI 94.72–100.00%) and a specificity of 89.55% (95% CI 87.76–91.16%) [89]. Further evidence suggests smartphone applications can potentially be used for remote monitoring of retinal pathology. The Checkup Vision Assessment System, a mobile app for iPhones, has demonstrated acceptable agreement with reference clinic readings for visual acuity and Amsler grid measurements. Importantly, patients reported high rates of usability and willingness to continue engaging with the mobile application [90]. Similar apps, such as the Alleye application for Android and iOS devices, have been evaluated against clinical outcomes. In a matched-pair analysis, eyes with macular pathology monitored at home with Alleye had greater visual acuity gains, fewer injection visits, and greater duration of follow-up [91].

### 6.3. Multidisciplinary Approach

A multidisciplinary approach will be required to improve the efficacy of DR screening. Initial evaluation of patients may be undertaken by an endocrinologist, optometrist, or PCP to reduce barriers to care. Recent literature has suggested that regular attendees of diabetic clinics are substantially more likely to participate in DR screening, highlighting the importance of such networks for recruitment [92]. Optometrists have been incorporated in such clinics to increase DR screening access for vulnerable populations [93], with the detection of DR demonstrated to be comparable between optometrists and ophthalmologists [93,94]. In locations without access to eye care specialists, placement of retinal imaging modalities within primary care or multispecialty clinics and/or robust integration of teleophthalmology services may further reduce obstacles for patients. The images acquired within the primary care setting are typically of sufficient quality for additional evaluation [95]. Teleophthalmology in primary care clinics via Intelligent Retinal Imaging Systems (IRIS) represents one financially sustainable option for increasing DR screening [58]. Teleophthalmology may additionally expand DR screening among diabetic patients in emergency departments [96]. Furthermore, with appropriate training, PCPs and endocrinologists can accurately screen fundus images to determine eyes that require ophthalmology referral [97,98]. Recent research has shown AI-based interpretation of fundus images to be a viable option for DR screening in primary care and endocrinology settings [99,100].

Educating patients about diabetic eye disease, including DR, is essential for preserving their vision. Unawareness of how diabetes leads to vision loss through the development of DR has been associated with lower screening rates [101,102]. Thus, patient education is a potent tool for enhancing disease prevention. In fact, individuals aware of diabetic eye disease are nine times more likely to attend DR screening clinics [103]. Similarly, increased DR screening rates are associated with patient education delivered via PCPs [104] and teleophthalmology screening [8]. Patient education may also be successfully delivered by dieticians and non-governmental organization (NGO) volunteers [93]. Promoting awareness of DR with a multidisciplinary approach will be crucial for effective teleophthalmology screening.

Cooperation between the public sector and private enterprise will also be important for the widespread implementation of DR screening, including services delivered by teleophthalmology. In an Indian DR screening program, NGO volunteers identified patients with diabetes, provided patient education, and raised awareness of a DR screening camp. Those diagnosed with DR at the camps were subsequently referred to a public tertiary care hospital [93]. Such initiatives could be further supported using government contracts with NGOs and other private organizations that already administer DR screening services [105]. Moreover, the costs of a DR screening program, including equipment, training personnel, quality assurance, and data security, will be substantial [105]. Cooperation between the public sector and private enterprises for the development and provision of these services will be necessary for successful and sustainable DR screening initiatives.

## 7. Conclusions

The increasing prevalence of DR presents issues with both screening and cost. The capacity of in-person screenings, typically provided by ophthalmologists or optometrists, may not be able to sustain pace with demand. As such, telemedicine in ophthalmology provides an opportunity to substantially expand screening capacity as well as to increase engagement for vulnerable populations. In concordance with these objectives, DR screening via teleophthalmology has been demonstrated to be accessible, cost-effective, and favorably perceived by patients. Accordingly, this technology, combined with a multi-disciplinary approach, could revolutionize the screening of DR. Nevertheless, barriers remain with deficiencies in technical implementation, provider training, and security hindering widespread adoption of teleophthalmology. Future directions include increasing funding for telemedicine, enhancing awareness of the necessity for DR screening, promoting multi-disciplinary cooperation, continuing investigation of economic models in teleophthalmology, and further developing AI-based healthcare platforms.

## Data Availability

No new data were created or analyzed in this study. Data sharing is not applicable to this article.
